# Metropolitan Hospital Sunday Fund

**Published:** 1908-08-08

**Authors:** 


### HOSPITAL ADMINISTRATION.

### CONSTRUCTION AND ECONOMICS.

### METROPOLITAN HOSPITAL SUNDAY FUND.

In the absence of the Lord Mayor, Archdeacon Sinclair presided at a meeting of the Council of the Metropolitan Hospital Sunday Fund at the Mansion House on Thursday, July 30.

Sir William Church reported the adoption of the following resolution : " That as there appears to be some doubt about the intention of the Council in passing their resolution relating to the Herring bequest on the 17th December last, which reads as follows :

'That in pursuance of the practice of Mr. Herring for some years, a sum of 5s. in the £ be added to the amount collected in places of worship in 1908 out of the money received from the Herring bequest,'

" the same be and is hereby rescinded, and that the amount of available income each year be added to the collections, and that the recommendations of the Committee of Distribution for the present year be adopted."

### DISTRIBUTION COMMITTEE'S REPORT.

The Committee of Distribution held its first meeting on Thursday, May 14, and at that and subsequent meetings examined the reports and income and expenditure accounts of the several institutions seeking to participate, and made their awards in conformity with Law V.

The executors of the late Mr. George Herring will pay to the treasurer of this Fund on July 30 £35,000, being the balance of income received for two years in respect of the existing investments after payment of certain expenses and previous charges.

The committee recommend that this sum be added to the collections, making the total of the Fund to date £72,386, in which is included a payment of £2,000 on account of the legacy of £10,000 receivable under the will of the late Mr. Herbert Lloyd. This year 259 institutions have applied for grants from the Fund, being seven more than in 1907. Three institutions were considered ineligible. The committee, while fully realising the excellent work carried on by the Cancer Hospital, have not made an award this year, for the reason stated in their report of 1906. The committee recommend that payment of the award to the Kensington General Hospital be deferred until the negotiations which are in progress between that institution and the King's Fund are satisfactorily concluded. The committee now recommend the distribution of £70,000 to the 164 hospitals and institutions, 60 dispensaries, and 29 nursing associations shown in the Appendix attached hereto.

The committee having reduced the bases of award in 14 cases, the fact was intimated to each of the hospitals interested in accordance with Law V. Ten deputations attended the committee, and after hearing their cases the final awards were determind.

The committee gather from a letter they have received from the Chairman of the West Ham Hospital that they propose to open the 40 additional beds they have provided. The committee fears that means for maintaining 40 additional beds may not be available in the present financial condition of the hospital.

Five per cent. of the total sum available for distribution is set aside for the purchase of surgical appliances during the ensuing year, and 2½ per cent. for district nursing associations.

In compliance with an order of the Council, and for the special use of its members, tables of statistics have been prepared as usual, showing an analysis of the number of beds in hospitals, the cost of patients at hospitals and dispensaries, the proportionate expense of management to maintenance, and other valuable information. A copy thereof has been sent to each member of the Council.

Sir William Church, in moving the adoption of the report, drew attention to the paragraph relating to the Kensington General Hospital. It was of very great importance that the three great funds should act in harmony and unison. The difficulty with this hospital was one of considerable standing. Some two or three years ago the King's Fund was willing to come to some arrangement, and would have obtained a hospital staff and put the institution on new lines. That, however, fell through, and the King's Fund was waiting till the hospital had a proper Committee and a proper staff arranged for.

Lord Cheylesmore seconded the resolution, but Dr. Glover did not consider the paragraph to which reference had been made a satisfactory one on the ground that such action might threaten the independence of the Sunday Fund. He thought they should make their own inquiries; indeed, their rules required it. He entirely sympathised with the idea that the different funds should work together. He knew nothing about the Kensington Hospital. They had always prided themselves on that Council on not interfering unduly with the internal arrangements of the institutions they assisted. He rather gathered that the question at issue was to do with the staff, and that brought them up against the question of medical qualification.

Sir William Church said the matter was one which had been under consideration for a very long time, and reminded Dr. Glover that the Kensington General Hospital used to be known as the Queen's Jubilee Hospital. He hoped the question would not now be pressed, but he should be pleased to go into it in detail at the office. The whole matter had been very carefully gone into by the Distribution Committee.

Dr. Glover adhered to his point, and moved that the award be not withheld except upon a report by the Distribution Committee after a separate investigation.

Prebendary Russell Wakefield said they had been given to understand by Sir William Church that such an inquiry had been made.

Sir Savile Crossley pointed out that the whole report of the Distribution Committee was based upon its own inquiries, but they had thought it better to mention this particular case.

Sir Henry Burdett emphasised the very great value of the principle of co-operation between the great funds which had been productive of more good in connection with hospitals than anything that had happened in the lifetime of anyone in that room. He hoped the Council would uphold the principle of co-operation.

The Chairman, having observed that a very useful discussion on an important point had been evoked, Dr. Glover withdrew his motion, and the adoption of the report was agreed to.

A vote of thanks to the Distribution Committee for the care bestowed on the preparation of the awards was moved by the Rev. E. H. Pearce, seconded by Canon Curtice, and adopted.

Sir Henry Burdett moved that the thanks of the Council be given to the editors of the newspapers who had pleaded the needs of the hospitals and advocated the cause of the Fund. He believed the newspapers might take an even more useful part in the work if they realised that the system on which the Sunday Fund worked was to concentrate their efforts on the week preceding Hospital Sunday. If the newspapers would co-operate to the extent of giving instructions to the sub-editors to see that each day in that week there was some paragraph referring to the hospitals or the Fund, and so keep the subject before the public, they would benefit greatly thereby, as was proved in 1895, when the collections amounted to £60,000. They had gone back in the year just past: it was a critical time in the City; but he was sure if they could only get from the editors of newspapers a little more organised support the collections next year would surprise them. But be that as it may, the Council were deeply grateful for the aid they had received. It was a cause which united all classes and all creeds, and one which must interest all newspaper readers.

Mr. John Hampton Hale seconded the resolution, which was adopted, and a vote of thanks to the Lord Mayor, moved by Sir Edward Durning Lawrence and seconded by the Rev. H. R. Gamble, brought the proceedings to a close.

### PARTICULARS OF THE AWARDS

### Recommended by the Committee of Distribution for the Year 1908

### THIRTY-FOUR GENERAL HOSPITALS.

Charing Cross Hospital, £1,530; French Hospital, £399 7s. 6d.; German Hospital, £708 15s.; Great Northern Central Hospital, £1,260; Guy's Hospital, £1,575; Hampstead General Hospital, £376 17s. 6d.; Italian Hospital, £225; Kensington General Hospital, £247 10s.; King's College Hospital, £1,800; London Hospital, £6,300; London Homœopathic Hospital £495; Phillips' Memorial Homœopathic Hospital, £51 15s.; London Temperance Hospital, £776 5s.; Metropolitan Hospital, £1,125; Mildmay Hospital, £337 10s.; Miller Hospital and Royal Kent Dispensary, £270; National Anti-Vivisection Hospital, nil; North-West London Hospital, £416 5s.; Poplar Hospital, £545 12s. 6d.; Tottenham, Prince of Wales' General Hospital, £736 17s. 6d.; Royal Free Hospital, £1,462 10s.; St. George's Hospital, £562 10s.; SS. John and Elizabeth Hospital, £371 5s.; St. John's Hospital, Lewisham, £196 17s. 6d.; St. Mary's Hospital, £2,610; St. Thomas's Hospital, £168 15s.; Seamen's Hospital Society, £1,665; the Middlesex Hospital and Convalescent Home, £2,700; University College Hospital, £2,553 15s.; Walthamstow, etc., Hospital, £151 17s. 6d.; Wandsworth, Bolingbroke Hospital, £326 5s.; West Ham Hospital, £601 17s. 6d.; West London Hospital, £1,276 17s. 6d.; Westminster Hospital, £1,428 15s.

### SPECIAL HOSPITALS.

Six Chest Hospitals.—City of London Hospital for Diseases of the Chest, Victoria Park, £900; Hospital for Consumption, Brompton, £3,420; Mount Vernon Consumption Hospital, Hampstead, £1,530; Royal Hospital for Diseases of the Chest, City Road, £613 2s. 6d.; Royal National Sanatorium (Bournemouth), £112 10s.; Royal National Hospital for Consumption (Ventnor), £337 10s.

Nineteen Children's Hospitals.—Alexandra Hospital for Hip Disease, W.C., £433 2s. 6d.; Banstead Surgical Home, £39 7s. 6d.; Barnet Home Hospital, £61 17s. 6d.; Belgrave Hospital for Children, S.W., £225; Cheyne Hospital for Incurable Children, S.W., £146 5s.; East London Hospital for Children, Shadwell, E., £1,006 17s. 6d.; Evelina Hospital for Sick Children, Southwark, S.E., £225; Home for Incurable Children, Hampstead, £84 7s. 6d.; Home for Sick Children, Sydenham, S.E., £171; Hospital for Sick Children, Great Ormond Street, W.C., £1,096 17s. 6d.; Kensington, for Children and Dispensary, £95 12s. 6d.; Paddington Green Hospital for Children, W., £337 10s.; Queen's Hospital for Children, Hackney Road, N.E., £1,001 5s.; Royal Waterloo Hospital for Children and Women, £202 10s.; St. Mary's Hospital, Plaistow, E., £326 5s.; St. Monica's Hospital, Brondesbury, N.W., £95 12s. 6d.; Victoria Hospital for Children, Chelsea, S.W., £843 15s.; Victoria Home, Margate, £45; Hospital for Hip Disease, Sevenoaks, £56 5s.

Eight Lying-in Hospitals.—British Lying-in Hospital, Endell Street, W.C., £119 5s.; City of London Lying-in Hospital, City Road, E.C., £200; Clapham Maternity Hospital, £45; East End Mothers' Home, £84 7s. 6d.; General Lying-in Hospital, Lambeth, S.E., £101 5s.; Plaistow Maternity Hospital, £67 10s.; Queen Charlotte's Lying-in Hospital, Marylebone Road, W., £579 7s. 6d.; Home for Mothers and Babies, Woolwich, £45.

Five Hospitals for Women.—Chelsea Hospital for Women, S.W., £343 2s. 6d.; Hospital for Women, Soho Square, W., £500 12s. 6d.; Grosvenor Hospital for Women and Children, Vincent Square, S.W., £208 2s. 6d.; New Hospital for Women, Euston Road, £399 7s. 6d.; Samaritan Free Hospital, Marylebone Road, W., £455 12s. 6d.

### TWENTY-FIVE OTHER SPECIAL HOSPITALS.

Cancer Hospital, Brompton, S.W., nil; Middlesex Hospital, Cancer Wing, £292 10s.; London Fever Hospital, Islington, N., £337 10s.; Gordon Hospital for Fistula, Vauxhall Bridge Road, S.W., £50 12s. 6d.; St. Mark's Hospital for Fistula, City Road, E.C., £292 10s.; National Hospital for the Diseases of the Heart, Soho Square, W., £191 5s.; Female Lock Hospital, Harrow Road, W., £281 5s.; Hospital for Epilepsy, Paralysis, and other Diseases of the Nervous System, Maida Vale, W., £236 5s.; National Hospital for the Paralysed and Epileptic, W.C., £1,091 5s.; West End Hospital for Diseases of the Nervous System, £472 10s.; Central London Ophthalmic Hospital, Gray's Inn Road, W.C., £196 17s. 6d.; Royal Eye Hospital, St. George's Circus, S.E., £360; Royal London Ophthalmic Hospital, City Road, E.C., £1,248 15s.; Royal Westminster Ophthalmic Hospital, Charing Cross, W.C., £185 12s. 6d.; Western Ophthalmic Hospital, Marylebone Road, W., £118 2s. 6d.; Royal National Orthopædic Hospital, Great Portland Street, W., £225; Royal Sea Bathing Hospital, Margate, £213 15s.; St. John's Hospital for Diseases of the Skin, £56 5s.; St. Peter's Hospital for Stone, Covent Garden, £135; Central London Throat and Ear Hospital, Gray's Inn Road, W.C., £19 2s. 6d.; Hospital for Diseases of the Throat, Golden Square, W., £16 17s. 6d.; London Throat Hospital, Great Portland Street, W., £28 2s. 6d.; Royal Ear Hospital, Dean Street, W., £51 15s.; Royal Dental Hospital of London, W.C., £416 5s.; National Dental Hospital, 149 Great Portland Street, W., £33 15s.

### THIRTY-THREE CONVALESCENT HOSPITALS.

Metropolitan Convalescent Institution, Walton, £478 2s. 6d.; Metropolitan Convalescent Institution, Broadstairs, £303 15s.; Metropolitan Convalescent Institution, Bexhill, £573 15s.; All Saints' Convalescent Hospital, Eastbourne, £450; All Saints' Convalescent Home, St. Leonards-on-Sea, £28 2s. 6d.; Ascot Priory Convalescent Home, £95 12s. 6d.; Brentwood Convalescent Home for Children, £11 5s.; Charing Cross Hospital Convalescent Home, Limpsfield, £73 2s. 6d.; Chelsea Hospital for Women Convalescent Home, St. Leonards, £50 12s. 6d.; Metropolitan Hospital Home, Cranbrook, £39 7s. 6d.; Deptford Medical Mission Convalescent Home, Bexhill, £20 5s.; Mrs. Gladstone's Convalescent Home, Mitcham, £95 12s. 6d.; Friendly Societies' Convalescent Home, Dover, £112 10s.; Hahnemann Convalescent Home, Bournemouth, £33 15s.; Hanwell Convalescent Home, £13 10s.; Hastings, Fairlight Convalescent Home, £31 10s.; Hendon, Ossulston Home, £64 2s. 6d.; Herbert Convalescent Home, Bournemouth, £22 10s.; Herne Bay Baldwin-Brown Convalescent Home, £67 10s.; Homœopathic Hospital Convalescent Home, Eastbourne, £16 17s. 6d.; Hemel Hempstead Convalescent Home, £31 10s.; Mrs. Kitto's Convalescent Home, Reigate, £56 5s.; London Hospital Convalescent Home, Tankerton, £81; Mary Wardell Convalescent Home for Scarlet Fever, £67 10s.; Police Seaside Home, Brighton, £50 12s. 6d.; St. Andrew's Convalescent Home, Clewer, £112 10s.; St. Andrew's Convalescent Home, Folkestone, £202 10s.; St. John's Home for Convalescent and Crippled Children, Brighton, £22 10s.; St. Joseph's Convalescent Home, Bournemouth, £56 5s.; St. Leonards-on-Sea Convalescent Home for Poor Children, £112 10s.; St. Mary's Convalescent Home, Shortlands, £25 17s. 6d.; St. Michael's Convalescent Home, Westgate-on-Sea, £39 7s. 6d.; Seaside Convalescent Hospital, Seaford, £168 15s.

### TWENTY-FIVE COTTAGE HOSPITALS.

Acton Cottage Hospital, £91 2s. 6d.; Beckenham Cottage Hospital, £92 5s.; Blackheath and Charlton Cottage Hospital, £90; Bromley, Kent, Cottage Hospital, £137 5s.; Bushey Heath Cottage Hospital, £42 15s.; Canning Town Cottage Hospital, £84 7s. 6d.; Chislehurst, Sidcup, and Cray Valley Cottage Hospital, £73 2s. 6d.; Coldash Cottage Hospital, £24 15s.; East Ham Cottage Hospital, £81; Eltham Cottage Hospital, £69 15s.; Enfield Cottage Hospital, £73 2s. 6d.; Epsom and Ewell Cottage Hospital, £45; Harrow Cottage Hospital (application withdrawn); Hounslow Cottage Hospital, £45; Livingstone Dartford Cottage Hospital, £108; Kingston, Victoria Hospital, £86 12s. 6d.; Mildmay Cottage Hospital, nil; Reigate and Redhill Cottage Hospital, £123 15s.; Sidcup Cottage Hospital, £45; Tilbury (Passmore Edwards) Cottage Hospital, £56 5s.; Willesden Cottage Hospital, £123 15s.; Wimbledon Cottage Hospital, £61 17s. 6d.; Wimbledon (South) Cottage Hospital, £66 7s. 6d.; Wood Green Cottage Hospital, £66 7s. 6d.; Woolwich and Plumstead Cottage Hospital, £56 5s.

### FIFTEEN INSTITUTIONS.

Hospital for Invalid Gentlewomen, Harley Street, £112 10s.; St. Saviour's Hospital and Nursing Home, £112 10s.; Invalid Asylum, Stoke Newington, £39 7s. 6d.; Firs Home, Bournemouth, £42 15s.; St. Catherine's Home, Ventnor, £22 10s.; Friedenheim Hospital for the Dying, £355 10s.; Free Home for Dying, Clapham, £126; St. Luke's House, Pembridge Square, W., £180; Santa Claus Home, Highgate, £68 12s. 6d.; Royal Mineral Water Hospital, Bath, £56 5s.; Winifred House, Holloway, £64 2s. 6d.; Eversfield, St. Leonards, nil; Infants' Hospital, Westminster, £112 10s.; All Saints', Highgate, £24 15s.

### SIXTY DISPENSARIES.

Battersea Provident Dispensary, £213 15s.; Billinsgate Dispensary, £118 2s. 6d.; Blackfriars Provident Dispensary, £22 10s.; Bloomsbury Provident Dispensary, £16 17s. 6d.; Brixton, etc., Dispensary, £56 5s.; Brompton Provident Dispensary, £19 2s. 6d.; Buxton Street Dispensary, £28 2s. 6d.; Camberwell Provident Dispensary, £90; Camden Town Provident Dispensary, £16 17s. 6d.; Chelsea, Brompton, and Belgrave Dispensary, £50 12s. 6d.; Chelsea Provident Dispensary, £11 5s.; Childs Hill Provident Dispensary, £16 17s. 6d.; City Dispensary, £56 5s.; Clapham General and Provident Dispensary, £28 2s. 6d.; Deptford Medical Mission, £28 2s. 6d.; Eastern Dispensary, £42 15s.; East Dulwich Provident Dispensary, £54; Farringdon General Dispensary, £45; Finsbury Dispensary, £63; Forest Hill Provident Dispensary, £42 15s.; Greenwich Provident Dispensary, £39 7s. 6d.; Hackney Provident Dispensary, £20 5s.; Hampstead Provident Dispensary, £69 15s.; Holloway and North Islington Dispensary, £22 10s.; Islington Dispensary, £68 12s. 6d.; Islington Medical Mission, £49 10s.; Kennington and Vauxhall Provident Dispensary, £15 15s.; Kensal Town Provident Dispensary, £16 17s. 6d.; Kentish Town Medical Mission, £23 12s. 6d.; Kilburn, Maida Vale, and St. John's Wood Dispensary, £45; Kilburn Provident Medical Institution, £58 10s.; London Dispensary, Spitalfields, £18; London Medical Mission, Endell Street, £153; Margaret Street, for Consumption, £34 17s. 6d.; Metropolitan Dispensary, £74 5s.; Mildmay Medical Mission Dispensary, £22 10s.; Notting Hill Provident Dispensary, £11 5s.; Paddington Provident Dispensary, £39 7s. 6d.; Public Dispensary, Drury Lane, W.C., £33 15s.; Queen Adelaide's Dispensary, £30 7s. 6d.; Royal General Dispensary, £30 7s. 6d.; Royal Pimlico Provident Dispensary, £54; Royal South London Dispensary, £51 15s.; St. George's, Hanover Square, Dispensary, £30 7s. 6d.; St. John's Wood Provident Dispensary, £57 7s. 6d.; St. Marylebone General Dispensary, £60 15s.; St. Pancras and Northern Dispensary, £36; St. Pancras Medical Mission, £24 15s.; South Lambeth, Stockwell, and North Brixton Dispensary, £43 17s. 6d.; Stamford Hill, Stoke Newington Dispensary, £63; Tower Hamlets Dispensary, £56 5s.; Walworth Provident Dispensary, £18; Wandsworth Common Provident Dispensary, £16 17s. 6d.; Westbourne Provident Dispensary, £22 10s.; Western Dispensary, £76 10s.; Western General Dispensary, £113 12s. 6d.; West Ham Provident Dispensary, £14 12s. 6d.; Westminster General Dispensary, £51 15s.; Whitechapel Provident Dispensary, £38 5s.; Woolwich Provident Dispensary, £38 5s.

### TWENTY-NINE NURSING ASSOCIATIONS.

Belvedere, Abbey Wood, £8 11s.; Brixton, £27 6s.; Central St. Pancras, £20 10s.; Chelsea and Pimlico, £27 6s.; Hackney, £34 4s.; Hammersmith, £54 14s.; Hampstead, £20 10s.; Isleworth, £13 13s.; Kensington, £61 10s.; Kilburn, £8 11s.; Kingston, £34 4s.; Metropolitan (Bloomsbury), £68 6s.; St. Olave's (Bermondsey), £47 16s.; Paddington and Marylebone, £47 16s.; Plaistow, £68 8s.; Plaistow (Maternity), £150 6s.; Rotherhithe, £13 13s.; Shoreditch £88 16s.; Silvertown, £12 16s.; South London (Battersea), £61 10s.; Tottenham, £6 16s.; Southwark, £41; South Wimbledon, £21 7s.; Westminster, £27 6s.; Woolwich, £29 18s.; East London, £177 13s.; North London, £75 3s.; London District, £492; Peckham, £8 11s.

